# Supplementary material for: Universal alignment in turbulent pair dispersion
Source: Nat Commun. 2023 Jul 14;14:4195. doi: 10.1038/s41467-023-39903-6 (PMC10345102; doi:10.1038/s41467-023-39903-6)
Supplement: Supplementary file 1 — Supplementary information [file 41467_2023_39903_MOESM1_ESM.pdf]

Supplementary information for: "Universal alignment in turbulent pair dispersion"

Ron Shnapp,[1,2] Stefano Brizzolara,[2,3] Marius M. Neamtu-Halic,[2,3] Alessandro Gambino,[2,3] and Markus Holzner[2,4]

[1] *Department of Mechanical Engineering, Ben-Gurion University of the Negev, Beer-Sheva, P.O.B. 653, Israel*
[2] *Swiss Federal Institute of Forest, Snow and Landscape Research WSL, 8903 Birmensdorf, Switzerland*
[3] *Institute of Environmental Engineering, ETH Zurich, CH-8039 Zürich, Switzerland*
[4] *Swiss Federal Institute of Aquatic Science and Technology Eawag, 8600 Dübendorf, Switzerland*

Supplementary information on pair dispersion

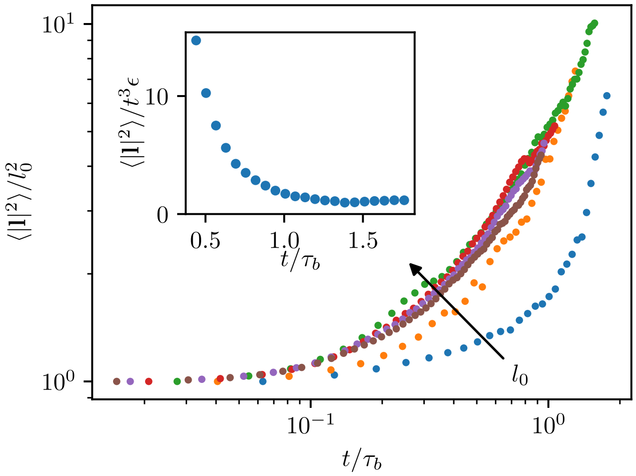

FIG. S1. The main panel shows the mean squared distance between pairs taken from the experimental data set. The data is normalized by the initial separation and plotted against time normalized by the Batchelor timescale. Data shown for bins with l_0/η spread between the borders 0, 4.5, 8, 14, 20, 25, and 30. The inset shows the mean squared distance between pairs normalized by $t^3\,\epsilon$ according to Richardson's scaling for $l_0 < 4.5\eta$.

To characterize pair dispersion in the experimental data set through the traditional framework, we plot the averaged squared distance between pairs against time in Fig.S1, where the abscissa is normalized by the Batchelor timescale and the ordinate is normalized by the initial separation. The pair dispersion curves have a dependence on the initial separation, as observed for the DNS dataset. The number of long trajectories with sufficiently small initial separation ($2\eta < l_0 < 4\eta$ was used for the DNS data shown in Fig. 2d of the main text) is not sufficient to robustly estimate g using the experimental results, although an approach toward a cubic scaling plateau can be identified in the inset of Fig. S1.

Figure S2 shows the probability distribution functions (PDF) of θ for the experimental data set, where the same results that were obtained using the DNS data set (Fig. 2e of the main text) are reproduced in the experiment. As time increases, the PDFs change their shape from a symmetric distribution relative to the central value of 90° to a strongly skewed distribution. This change in shape

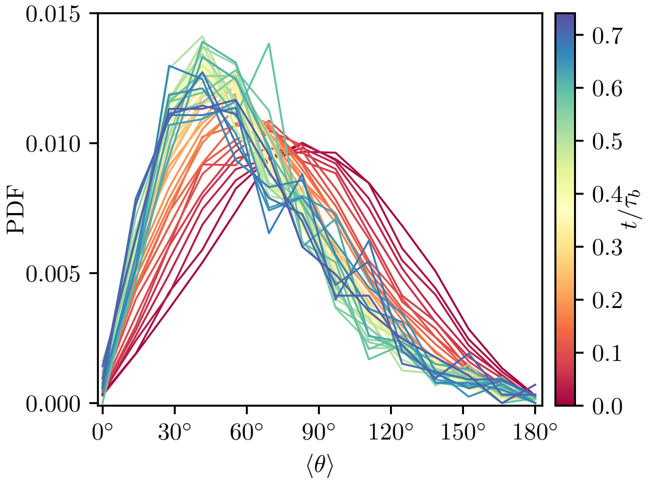

FIG. S2. Probability distribution functions for the average angle $\langle\theta\rangle$ plotted at various times for pairs with $16.5\eta < l_0 < 21\eta$ from the experimental data set.

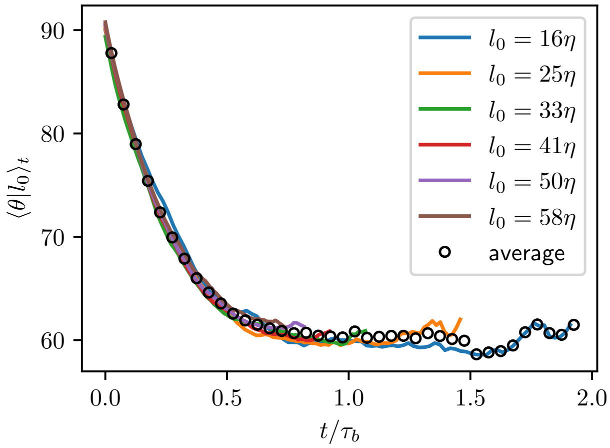

FIG. S3. The angle θ, calculated from the experimental data set, and averaged over trajectory groups based on l_0, is plotted against the time normalized by the Batchelor timescale. The trajectories are divided into groups based on l_0 with a width of 8η centered around the values shown in the legend of the figure. The average across the groups at fixed t/τ_b values is shown as circles.

corresponds to the oblique values of $\langle\theta\rangle$ at times longer than approximately $0.5\tau_b$ that are shown in Fig.3a of the paper. In addition, Fig. S3 shows the good collapse of

$\langle\theta\rangle$ from various initial separations in the inertial range in the experiment. The circles correspond to the average across l_0, which is also shown in the paper (Fig. 3a inset).

Code for downloading data from the JHTDB

The DNS dataset we used was downloaded from the Johns Hopkins Turbulence Database (JHTDB). We downloaded data from the data base in groups of `Np` particles each time. Each group central position was chosen randomly in the domain at a specified initial time instance. The individual particles were also scattered around the center randomly. The trajectories were integrated for a duration of three integral timescale during which 200 time steps were saved. To integrate trajectories and download the data set we used the get position function, while storing the data on the disk every five time steps as a backup against errors. The data for each group was stored in a zip archive using the `Numpy` format. The data was downloaded using the Python code provided below.

```
import numpy as np
import os
import pyJHTDB
import pyJHTDB.dbinfo

lJHTDB = pyJHTDB.libJHTDB()
lJHTDB.initialize()

auth_token  = "*place your token here*"
lJHTDB.add_token(auth_token)

fname = 'FileName.npz'

# particle number
Np = 200

# random center point:
P = 2*np.pi * np.random.random(size=3)

# random cloud around it:
eta_k = 0.00287
R = (eta_k * 1.0)
X0 = P + R * np.random.random((Np, 3))

# times
T = 3.0
t0 = 2.0
t_end = t0 + T

# steps
steps = 200

# step saving:
ds = 5

ls = os.listdir('./')
if fname in ls:
        raise ValueError('fname already exists in the folder.')

s = 0
while s< steps:

        print('%d / %d'%(s,steps))

        if s == 0:
                x0 = X0
```

```
            t0_ = t0

    else:
            data = np.load(fname)
            x_past = data['x']
            t_past = data['t']
            x0 = x_past[-1, : :]
            t0_ = t_past[-1]

    x, t = lJHTDB.getPosition(
                    starttime = t0_,
                    endtime = t0_ + ds/steps*(t_end - t0),
                    dt = 0.001,
                    point_coords = x0 ,
                    steps_to_keep = int(ds))

    if s==0:
            np.savez(fname, x=x, t=t)

    else:
            X_updated = np.append(x_past, x[1:,:,:], axis=0)
            t_updated = np.append(t_past, t[1:], axis=0)
            np.savez(fname, x=X_updated, t=t_updated)

    s+=ds

lJHTDB.finalize()
print('\n','finished!')
```
