# Supplementary material for: Universal alignment in turbulent pair dispersion
Source: Nat Commun. 2023 Jul 14;14:4195. doi: 10.1038/s41467-023-39903-6 (PMC10345102; doi:10.1038/s41467-023-39903-6)
Supplement: Supplementary file 2 — Description of Additional Supplementary Files [file 41467_2023_39903_MOESM2_ESM.pdf]

<u>Description of Additional Supplementary Files</u>

Supplementary Video 1: A 3D rendered visualization of the trajectories of two particles from the DNS data set. The relative position and relative velocity direction vectors are visualized by lines, and the pair dispersion angle between the mis shown as well. The shade of the lines along the trajectories corresponds to the value of the pair dispersion angle.

Supplementary Video 2: A 3D rendered visualization of particle trajectories from the experimental dataset. The video corresponds to one second of recorded data, and the speed of each trajectory is indicated by the shades of the trajectory points.
